# Single-cell analysis reveals significant transcriptomic alterations in preclinical Crohn’s disease

**DOI:** 10.3389/fimmu.2025.1539830

**Published:** 2025-05-22

**Authors:** Dimitrios Kioroglou, Leire Egia-Mendikute, Asis Palazon, Manuel Barreiro-de Acosta, Iago Rodríguez-Lago, Urko M. Marigorta

**Affiliations:** ^1^ Integrative Genomics Lab, Center for Cooperative Research in Biosciences (CIC bioGUNE), Basque Research and Technology Alliance (BRTA), Bizkaia Technology Park, Derio, Spain; ^2^ Cancer Immunology and Immunotherapy Lab, Center for Cooperative Research in Biosciences (CIC bioGUNE), Basque Research and Technology Alliance (BRTA), Bizkaia Technology Park, Derio, Spain; ^3^ IKERBASQUE, Basque Foundation for Sciences, Bilbao, Spain; ^4^ Gastroenterology Department, Hospital Clínico Universitario de Santiago, Santiago de Compostela, Spain; ^5^ Gastroenterology Department, Hospital Universitario de Galdakao, Biobizkaia Health Research Institute, Galdakao, Spain; ^6^ Department of Medicine, Faculty of Health Sciences, University of Deusto, Bilbao, Spain

**Keywords:** Crohn’s disease, scRNAseq, PBMC, preclinical, IBD

## Abstract

Despite the increasing incidence of Crohn’s disease (CD), its early immune disturbances have not all been described yet. We analysed single-cell RNASeq data from peripheral blood mononuclear cells of patients with incidentally-diagnosed CD, and compared their gene expression profile to healthy individuals. The main aim of our study was to perform an exploratory analysis of how the subclinical inflammatory process is modifying the individual’s immunologic environment while the patient is still in the preclinical period.

## 1 Introduction

Crohn’s disease (CD) and ulcerative colitis are chronic immune-mediated inflammatory diseases that consist the two primary forms of IBD. Due to the increasing worldwide incidence of IBD, the study of its preclinical phase is crucial since it will allow the understanding of the underlying immunological pathways that lead to the development of the disease prior to its clinical onset. Various initiatives have focused on providing insights into the transitional phase of the disease, such as the Genetics Environmental Microbial (GEM) project (1) and the Proteomic Evaluation and Discovery (PREDICTS) project (2). Moreover, a recent review by Rudbaek et al. (3) provided numerous studies that investigated the preclinical phase of IBD, either at a dedicated time-point or longitudinally, deriving insights from diverse omic samples such as serological proteomics and metabolomics as well as intestinal microbiome. Although these studies have demonstrated the possibility to identify processes that can be linked to the development of IBD well in advance of the clinical onset, still the transitional events that shift the asymptomatic patient towards the symptomatic phase remain unknown.

The EARLY study (NCT05698745) represents our prospective 10-years planned initiative, in collaboration with 25 hospitals in Spain, that will enrol asymptomatic IBD patients who have been incidentally diagnosed through general colorectal cancer screening programs (4). By implementing multi-omic integration and drawing comparisons between asymptomatic and symptomatic IBD patients, the goal of EARLY is to pinpoint the initial immunological alterations that modify the immunologic environment of patients during the preclinical phase of IBD and favor the transition towards the clinical onset of the disease. As part of EARLY, the current manuscript represents a pilot study of single-cell RNA sequencing (scRNASeq) from peripheral blood mononuclear cells (PBMC) of two preclinical CD patients, where their gene expression profile was compared to two non-IBD healthy individuals. Our aim was to explore the potential differences between the two groups, since characterization of the molecular landscape at the single-cell level is still lacking during the preclinical stage.

## 2 Methods

PBMC samples from two CD patients were collected within the first 2 months after diagnosis and without previous IBD-related therapy. These two patients included a 52-year-old female patient with ileocecal disease (L3) and inflammatory behaviour (B1), former smoker, and a 54-year-old male with ileal involvement (L1) and also inflammatory behaviour (B1), non-smoker. Neither of these patients had disease-related complications or extraintestinal manifestations. They were incidentally (i.e. asymptomatic) diagnosed with CD within the population-based colorectal cancer screening programme of the Basque Country (https://www.osakidetza.euskadi.eus/programa-cribado-cancer-colorrectal/webosk00-oskenf/es/). Both patients were asymptomatic at diagnosis, which was made according to ECCO criteria, during a complete screening colonoscopy after a positive fecal occult blood test (cut-off 20 *µ*g Hb/g). Clinical, endoscopic and histologic findings confirmed the diagnosis of CD by the presence of chronic infiltrate and absence of any enteropathogen or alternative diagnosis after a detailed differential diagnosis. This methodology has been used in previous reports by our group (4–6) and recognized by international consensus as a potential target for disease intervention studies (7).

The frozen vials of PBMCs were rapidly thawed in a 37°C water bath for 2 min and diluted in 4 mL of DMEM medium (Gibco # 41966). Cells were centrifuged at 500 x g for 10 min at room temperature. The supernatant was removed, and the cell pellet was resuspended in 3 mL of cold 1X phosphate-buffered saline (PBS, Thermo Fisher Scientific). Live cells were sorted by FACSfusion using DAPI. Cells were resuspended with PBS at a concentration of 1.000 cells/mL for single-cell RNA sequencing (scRNAseq).

Single cell library was prepared using Chromium Next GEM Chip G Single Cell Kit, Chromium Next GEM Single Cell 3’ Kit v3.1 and Dual Index Kit TT Set A, following Chromium Next GEM Single Cell 3’ Reagent Kits v3.1 (Dual Index) user guide. The sequencing process was carriedout on a NovaSeq 6000 sequencer, whereas alignment and gene count was performed using 10X Genomics Cell Ranger 7.0.0 and the human reference GRCh38.

For the control dataset, two healthy non-IBD controls were selected from two distinct PBMC scRNAseq datasets, after reassuring that each sample underwent the same 10X Genomics 3’ V3 protocol that was implemented in our study. The first sample was selected from the publicly available PBMC scRNAseq control dataset provided by 10X Genomics (8) and the second sample was an untreated healthy individual selected from the publicly available scRNAseq dataset of PBMCs (1M-scBloodNL) provided by Oelen et al. (9).

We performed the following quality control to each sample individually using SCANPY (10). We filtered out genes with gene count <10 across all cells. We removed cells with high mitochondrial content and removed mitochondrial genes from the list of quantified genes. We normalized total counts per cell to 10,000. We calculated cell-cycle scores using the “scanpy_usage” github repository (https://github.com/scverse/scanpy_usage) to score S and G2M phases and removed cycling cells. We predicted and removed doublets using SCRUBLET (11). We finally performed cell-type annotation using AZIMUTH (12) that utilized the AZIMUTH human PBMC reference dataset (13).

We next merged the samples and performed batch correction with SCVI-TOOLS (14) in order to correct data integration from different studies. Finally, for each cell-type we performed differential gene expression analysis with SCANPY using the batch corrected gene expression estimated by SCVI-TOOLS. Using the identified differential expressed genes, we performed pathway enrichment analysis and functional annotation. Enrichment analysis was performed with the Cytoscape plug-in ClueGO (15) where databases from KEGG, Reactome and WikiPathways were queried for pathway enrichment and annotation. For the functional annotation of proteins we utilized the PANTHER API (16).

## 3 Results

**Figure 1:**
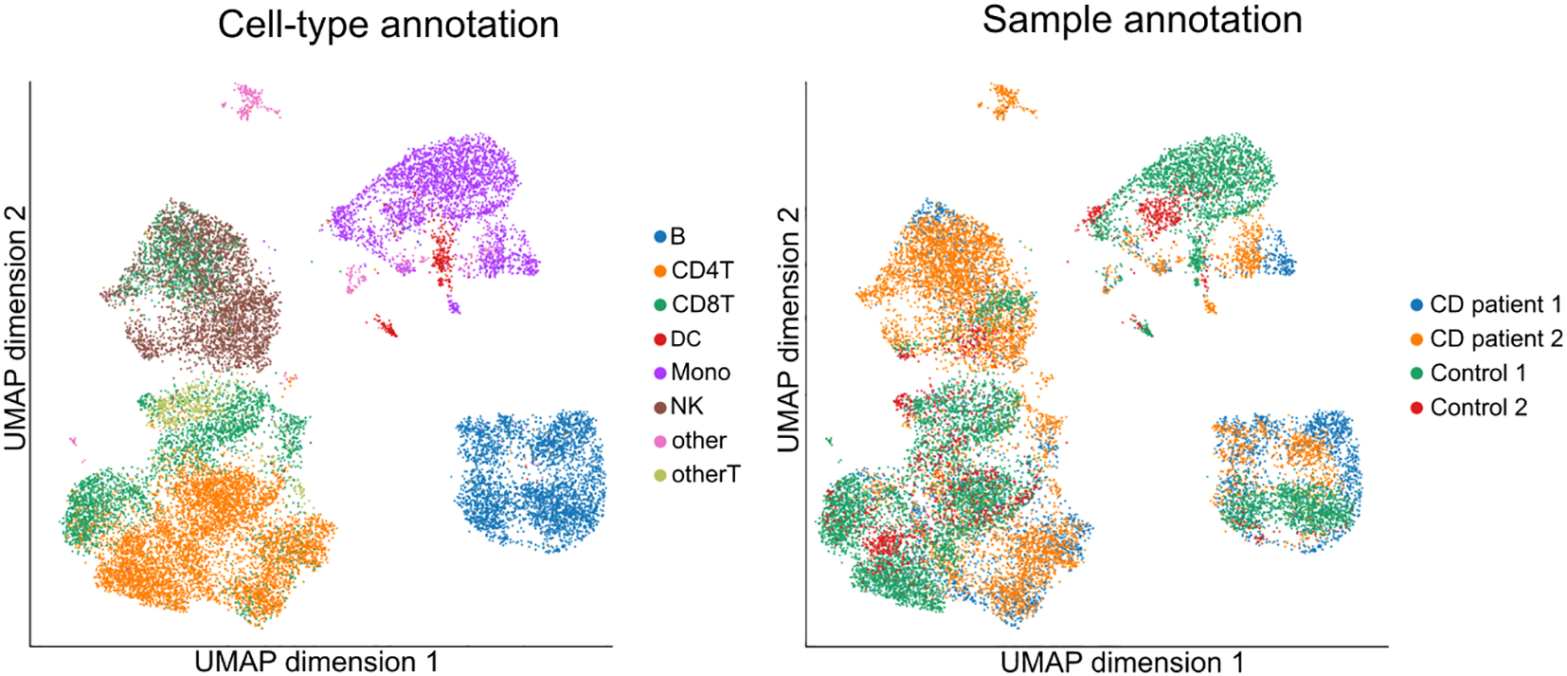
Uniform manifold approximation and projection (UMAP) of cells annotated by celltype (left panel) and sample (right panel).

Initially, we evaluated the pre-processing pipeline that included the merging of the datasets. We did not observe formation of separate clusters between the samples for the same cell-types **Figure 1** right panel), indicating successful batch correction concerning integration from different studies Luecken et al. (17). The cell-type annotation identified B cells (B), CD4^+^ and CD8^+^ T-cells (CD4T, CD8T), dendritic cells (DC), monocytes (Mono) and natural killer cells (NK) (**Figure 1** left panel). The analytical pipeline included 17,021 cells and 3,000 quantified genes. Considering each cell type individually, we identified 49 differentially expressed genes (DEG) between CD patients and controls, from which 30 were DEGs in all cell types.

**Figure 2:**
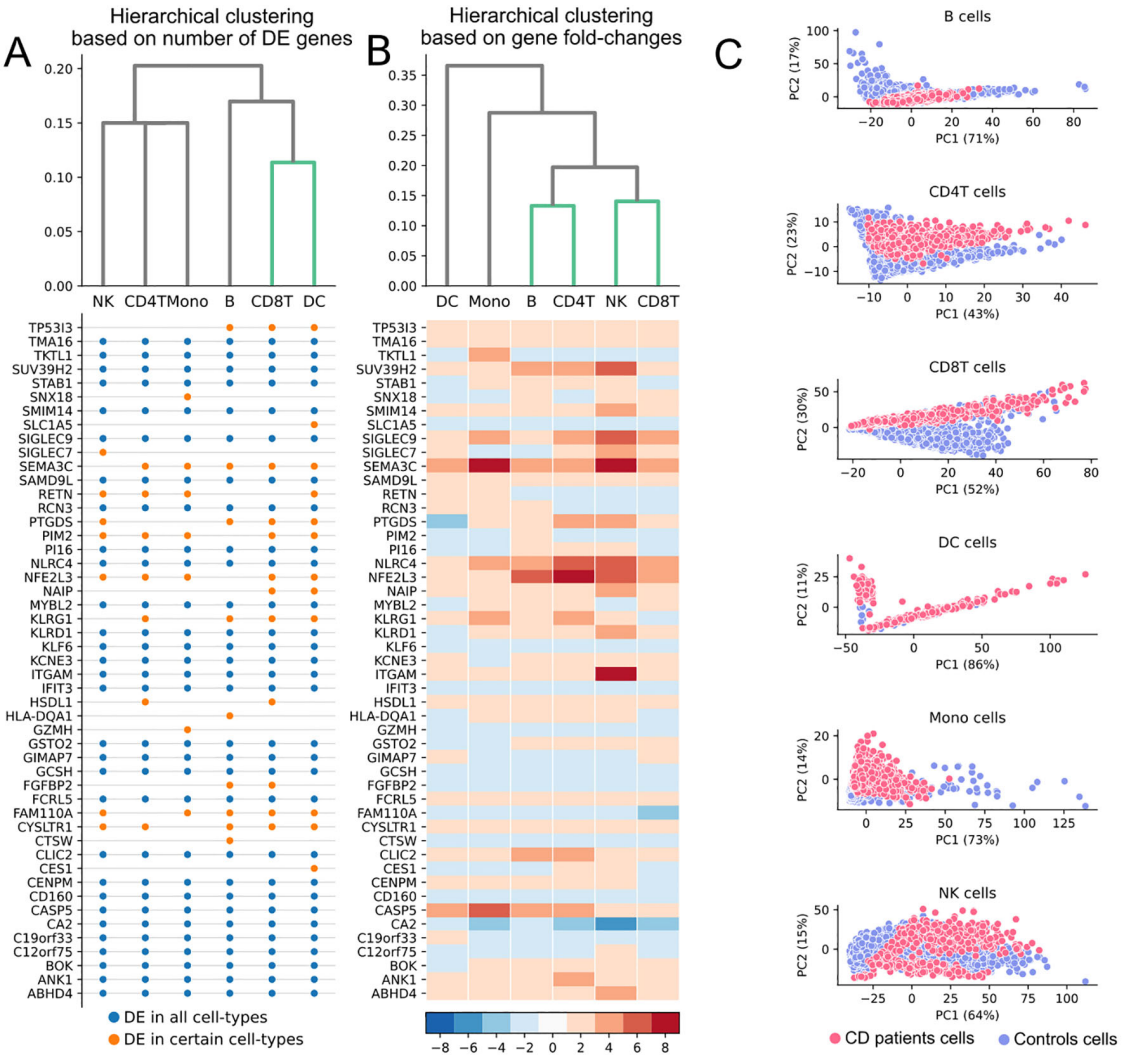
Clusterings based on 49 DEGs identified between CD patients and controls. **(A)** Hierarchical clustering based on the fraction of the 49 DEGs that were differentially expressed in each cell-type. **(B)** Hierarchical clustering based on the fold-changes of the 49 DEGs between CD patients and controls in each cell-type. **(C)** PCA using the 49 DEGs in each cell-type.

Building on this list of 49 detected DEGs, we report four insights into the preclinical period of the disease. First, we observed greater similarity between CD8T and DC cells since they were the cell types with higher fraction of DEGs (**Figure 2A**). This suggests a higher contribution of these cell-types in the separation of the two groups. On the other hand, gene fold-changes between the two groups showed similar expression patterns between the CD4T and B cells as well as between CD8T and NK cells (**Figure 2B**).

**Figure 3:**
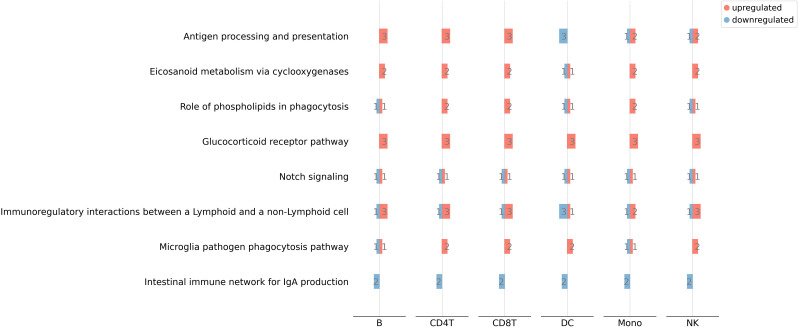
Pathway enrichment analysis of 30 genes that were differentially expressed across all cell types.

Second, pathway analysis revealed significant enrichment of the pathway that is related to immunoregulatory interactions between lymphoid and non-lymphoid cells (**Figure 3**). Five genes belonged to this enriched pathway, namely *CD160*, *KLRD1*, *KLRG1*, *SIGLEC7* and *SIGLEC9*. All cell-types displayed significant alterations in the mRNA levels of *CD160*, *KLRD1* and *SIGLEC9*, whereas *SIGLEC7* was differentially expressed only in NK cells, and *KLRG1* in all cells except NK and Mono cells. Furthermore, functional annotation analysis identified the gene products of *FCRL5*, *KLRD1*, *CES1*, *HLA-DQA1*, *IFIT3*, *PI16* as proteins related to immunological defense.

Third, we identified significant transcriptomic alterations in genes previously related to the symptomatic phase of IBD, or reported as potential biomarkers. The differentially expressed *FCRL5* and *PIM2* have been reported as significantly upregulated in plasma cells of mesenteric adipose tissue of CD patients (18). We observed significant downregulation of *CD160* that has been related to the development of the IBD pathogenesis (19). The gene *CA2* has been identified as a biomarker in ileal CD (20) and it was differentially expressed in all cell-types in the current study. Whole blood transcriptomic data analysis included *CYSLTR1* among the 100 genes that could be potentially associated with IBD (21), and it was differentially expressed and upregulated in almost all cell-types of CD patients in our study. The serum levels of *NLRC4* have been reported significantly lower in CD patients using enzyme-linked immunosorbent assay (22). Although *NLRC4* was differentially expressed across all cell-types in our study, we observed upregulated levels in the CD patients. Additionally, it has been reported that the activation of *NLRC4* is mediated by *NAIP* (23) which was differentially expressed and upregulated in the CD8T and DC cells of the CD patients in the current study.

Fourth, principal component analysis (PCA) using the 49 DEGs showed high captured variance with the first two PCs in all cell-types (**Figure 2C**). Of note, we observed greater separation between patients and controls in the CD8T cells. The separation occurred mainly across the PC2 that captured the highest variance in the CD8T cells compared to the other cell-types. The genes that exhibited strong negative correlation (≤-0.8) with the PC2 and were differentially expressed in CD8T cells were *KLRD1*, *ITGAM*, *SMIM14*, *SAMD9L*, *PTGDS*, *KLRG1*, whereas the genes *GIMAP7*, *KLF6*, *FGFBP2* showed strong positive correlation (≥0.8). Interestingly, *KLRD1* and *KLRG1* have been suggested to be among the targets of the T-box transcription factor Eomesodermin (Eomes) during the shifting of CD8^+^ tissue-resident memory T cells towards an inflammatory differentiation state in UC patients (24). Thus, capturing such transcriptomic events in the preclinical phase highlights the potential role of CD8 T-cells during the initial events of the immunopathogenesis of CD. This hypothesis is inline with previous studies, postulating that the transcriptomic profile of this cell-type is able to predict a more frequently relapsing disease (25). Additionally, the serum protein levels of *CXCL9*, which is involved in T-cell trafficking, have recently been found significantly associated with the future manifestation of CD pathogenesis (26).

## 4 Discussion

IBD represents a chronic and progressive disease with a complex pathophysiology. Although the immunological events that drive its pathophysiology remain unknown, various studies hint their presence years before the onset of the first symptoms (3). In addition, life style and environmental factors make their own contribution to the disease onset, modulating its progression and increasing its complexity. Thus, longitudinal studies that integrate multi-omic data are necessary to study the transition of IBD patients from the preclinical phase.

Our pilot study is the first that incorporates PBMC single-cell expression data of CD asymptomatic patients. Overall, we observed distinct transcriptomic profiles of these patients compared to healthy individuals, with small intragroup differences. Of note, although correction methods were applied to minimize technical biases, differences in sequencing platforms, sample processing, and population structure between datasets may still introduce residual confounding effects, which we acknowledge as a limitation of the study.

Nevertheless, our preliminary results revealed significant transcriptomic alterations in previously reported genes and highlighted CD8T cells as the cell-type that has greater impact on the separation of the two groups. In a systematic review conducted by Chen et al. (27), subsets of CD8T populations have exhibited prognostic potential to predict either post-operative endoscopic recurrence, risk of surgery or future response to vedolizumab treatment. Thus, the emergence of patterns associated with CD8T cells in our preliminary results are encouraging findings and evoke anticipation for larger sample size attempts towards characterization of the preclinical stage of IBD, such as the EARLY study, that will serve to validate the first principles described here.

## 5 Conclusion

Our preliminary findings directly support our aim of exploring how subclinical inflammation shapes the immunologic landscape during the preclinical phase of CD. The identification of distinct transcriptomic profiles compared to healthy controls and the prominent role of CD8T cells suggest that certain immunologic alterations can be detectable during the asymptomatic phase. Thus, providing the first single-cell approximation, our pilot study adds novel transcriptomic insights that complement the emerging picture about the molecular changes that precede the onset of symptomatic IBD and paves the way for the discovery of new markers allowing the development of potential disease intervention strategies at an early stage in this disease.

## Data Availability

The datasets presented in this study can be found in online repositories. The names of the repository/repositories and accession number(s) can be found below: https://www.ncbi.nlm.nih.gov/sra/PRJNA970312, PRJNA970312.
